# The Evaluation of Counter Diffusion CVD Silica Membrane Formation Process by In Situ Analysis of Diffusion Carrier Gas

**DOI:** 10.3390/membranes12020102

**Published:** 2022-01-18

**Authors:** Katsunori Ishii, Mikihiro Nomura

**Affiliations:** Department of Applied Chemistry, Shibaura Institute of Technology, 3-7-5 Toyosu, Koto-ku, Tokyo 135-8548, Japan; na19101@shibaura-it.ac.jp

**Keywords:** silica membrane, counter diffusion CVD, deposition rate, mass spectroscopy, silica precursor

## Abstract

A new evaluation method for preparing silica membranes by counter diffusion chemical vapor deposition (CVD) was proposed. This is the first attempt to provide new insights, such as the decomposition products, membrane selectivity, and precursor reactivity. The permeation of the carrier gas used for supplying a silica precursor was quantified during the deposition reaction by using a mass spectrometer. Membrane formation processes were evaluated by the decrease of the permeation of the carrier gas derived from pore blocking of the silica deposition. The membrane formation processes were compared for each deposition condition and precursor, and the apparent silica deposition rates from the precursors such as tetramethoxysilane (TMOS), hexyltrimethoxysilane (HTMOS), or tetraethoxysilane (TEOS) were investigated by changing the deposition temperature at 400–600 °C. The apparent deposition rates increased with the deposition temperature. The apparent activation energies of the carrier gas through the TMOS, HTMOS, and TEOS derived membranes were 44.3, 49.4, and 71.0 kJ mol^−1^, respectively. The deposition reaction of the CVD silica membrane depends on the alkoxy group of the silica precursors.

## 1. Introduction

Membrane separation technology is an energy-saving technology with no phase transition. Developing gas separation membranes is key for a carbon-free society. Several separation targets exist for the production and purification of hydrogen (H_2_) [1,2], carbon dioxide recovery [3], and olefin/paraffin separation [4]. Various types of membranes are developed by using suitable components, such as palladium alloy, zeolite, silica, carbon, and metal organic frameworks for the separation targets. Such membranes are effective in separation as well as reaction-separation processes, thus being developed as membrane reactors.

Amorphous silica membranes showing H_2_ permselectivity, are promising candidates for separation membranes and membrane reactors. An ideal membrane must contain no defects, be highly permeable to the target substance, and exhibit high separation performance. Silica membranes are developing several methods for becoming thinner and more homogeneous. Several methods, including the sol-gel method [5,6] and chemical vapor deposition (CVD) [7,8,9,10,11,12] have been developed for thin and homogenous silica membrane fabrication. In the sol-gel method, silica precursor is gelled in the liquid phase by polycondensation, and a thin membrane is obtained by dip or spin coating the substrate and gelling it by calcination. In the CVD method, the silica precursor vapor is activated by heat [7], oxidant [8,9,10], or plasma [11,12], etc., and deposited on the porous substrate.

**Figure 1:**
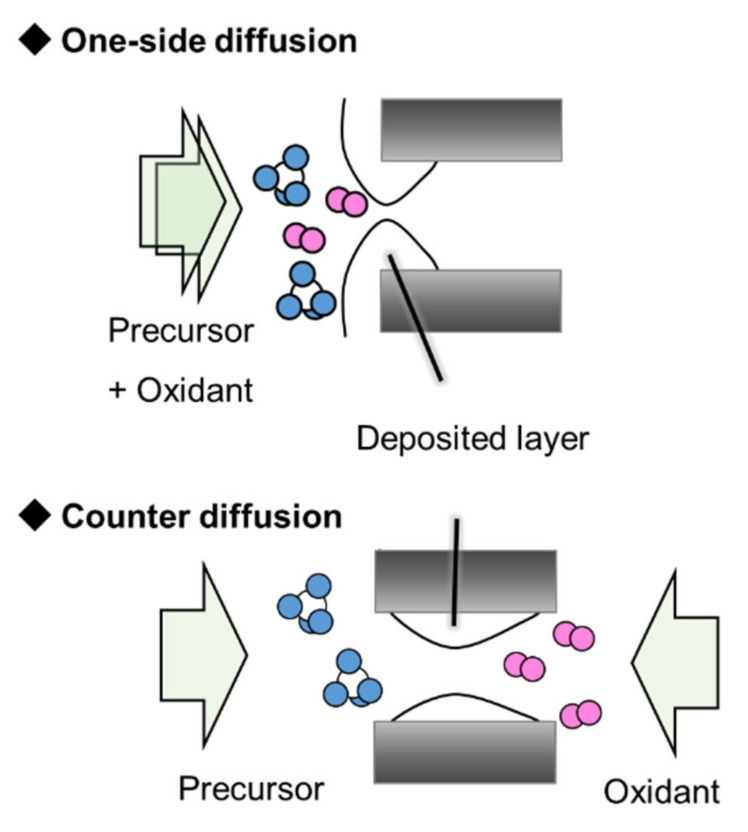
Schematic image of membrane preparation method by supplying reaction products [13,14].

In this study, the CVD silica membranes developed as membrane reactors are focused on. The deposition reaction can be classified by the geometry suppling the reactant(s) (Figure 1). One side of the diffusion CVD involves supplying the precursor (and oxidant) from the same side of the substrate [13]. The other side is supplied with sweep gas or vacuumed to diffuse the reactant(s) into the substrate pores. Counter diffusion CVD is another method for preparing silica membrane. The precursor and the oxidant are supplied to the opposite sides of the substrate [14]. The reactants diffuse into the substrate pores through the partial pressure difference. The deposition reaction occurs in the pores of the substrate and is automatically stopped by suppressing the reactant diffusion by depositing silica in the pores. Diffusion of the reactive species is more likely to occur in the region with the larger pore size, and a uniform silica membrane can be obtained.

The CVD silica membranes are developed as the H_2_ separation membrane and the membrane reactor to improve the dehydrogenation reaction conversion. Gavalas et al. [8] first reported the counter diffusion CVD treatment using monosilane/oxygen (O_2_)/nitorogen (N_2_) for controlling the fine pores of the Vycor glass tube substrate with about 4nm pores. The membrane showed H_2_ permselectivity with the H_2_/N_2_ gas permeance ratio of 3300 [8]. Monosilanes [8], tetrachlorosilanes [13], and tetraethoxyorthosilicates (TEOS) [9] have been used as precursors, but easy-to-handle alkoxysilanes are often reportedly used as well. Yamaguchi et al. [10] developed silica membrane using ozone (O_3_) as an oxidant for the counter diffusion CVD treatment at 175–300 °C. Tetramethoxyorthosilicate (TMOS) and TEOS with different alkoxy groups were used as precursors. The TMOS-derived membrane showed a helium (He)/N_2_ permeance ratio of 840. Nakao et al. reported the He/N_2_ permeance ratio of 950 by using TEOS and ozone as the reactants deposited at 200 °C [15]. Nomura et al. reported TMOS-derived membrane prepared by counter diffusion CVD treatment at 600 °C with the He/N_2_ permeance ratio of 5700 [14]. The silica membranes can be used in membrane reactors because of their ability of extracting H_2_ to improve the dehydrogenation reaction conversion. The catalyst composite membrane reactor was proposed to extract H_2_ for the steam methane reforming reaction [16]. The conversion at 500 °C improved from 31.4% to 64.5% by H_2_ extraction using silica membranes. The reaction conversion can be improved by extracting hydrogen with reports on ethane dehydorogenation [17], propane dehydrogenation [18], and methylcyclohexane dehydrogenation [19]. The larger the H_2_ permeance of the membrane, the more it improves the membrane reactor.

Generally, the silica membrane pore size is about 0.3 nm because of the H_2_ (0.29 nm) permselectivity. Recently, control of the silica membrane pore size has also been studied by using alkylalchoxysilane as a precursor. Sea et al. [20] prepared the membrane by using the silica precursor of TEOS, phenyltriethoxysilane (PhTEOS), or diphenyldiethoxysilane (DPhDEOS) for the one-diffusion CVD method at 500–600 °C. The TEOS-derived membrane showed an H_2_/N_2_ permeance ratio of 43 at 200 °C. Conversely, the PhTEOS- or DPhDEOS-derived membranes showed an N_2_ (0.36 nm)/sulfur hexafluoride (SF_6_, 0.55 nm) permeance ratio of 11 or 18 at 200 °C, indicating that the pore size is larger than that of the TEOS-derived membrane. Ohta et al. [21] reported the effects of the silica precursor of TMOS, phenyltrimethoxysilane (PhTMOS), and diphenyldimethoxysilane (DPhDMOS) for the counter diffusion CVD method. The DPhDMOS-derived membrane showed a N_2_/SF_6_ permeance ratio of about 50 at 300 °C. Nomura et al. [22] reported the effects of a precursor of TMOS, methyltrimethoxysilane, propyltrimethoxysilane (PrTMOS), or PhTMOS on the permeation performance for the counter diffusion CVD method. The TMOS-derived membrane showed an H_2_/N_2_ permeance ratio of about 500 with an H_2_ permeance of 9.1 × 10^−8^ mol m^−2^ s^−1^ Pa^−1^, while the PrTMOS-derived membrane showed a lower H_2_/N_2_ permeance ratio of 30 with higher a N_2_/SF_6_ permeance ratio of 30. The precursors with methyl [23], ethyl [18,24], propyl [24,25], butyl [18,23], hexyl [1,18,24,25,26,27], phenyl [28,29], 3-aminopropyl [23], and 3,3,3-trifluoropropyl [30] groups were investigated. Matsuyama et al. [25] prepared the silica membrane via CVD using hexyltrimethoxysilane (HTMOS) as a precursor. The membrane deposited for 5 min at 450 °C showed a propylene (C_3_H_6_)/propane (C_3_H_8_) permeance ratio of 364 with a pore size of about 0.47–0.50 nm considering the molecular sizes of C_3_H_6_ and C_3_H_8_. Myagmarjav et al. [1,26,27] developed an H_2_ permselective membrane reactor to improve the conversion of the hydrogen iodine (HI) decomposition reaction for the iodine–sulfur process. To improve the H_2_ permeance, HTMOS is used as a precursor to obtain larger pores, because of the molecular size of HI being 0.55 nm, which is larger than that of N_2_. The durability of HI vapor was tested for 11 h. HI permeance was stable with a very low permeance of about 4.3 × 10^−10^ mol m^−2^ s^−1^ Pa^−1^ and a H_2_/HI gas permeance ratio of 1296 at 400 °C. Ikeda et al. [24] reported the effect of alkyl groups of the silica precursors on the deposited silica for separating hydrocarbons. A methane/ethane permeance ratio of 10 was found for the ETMOS-derived membrane. These types of membranes succeeded in separating liquids, including sodium chloride [28], sulfuric acid [29], glucose, magnesium chloride, and sodium sulfate aqueous solution [30].

As described above, there are abundant precursor and deposition conditions for developing silica membranes. Various separation investigations have been conducted for the same. However, the reaction mechanism has not be discussed sufficiently. Silica deposition reaction mechanism is only investigated using TEOS thermal decomposition [31], TEOS/O_2_ [32,33]_,_ or TEOS/O_3_ deposition [34] for preparing for nonconductor application in the semiconductor field. Reaction was evaluated by stacking properties of the silica layer in a micro-trench. The apparent reaction kinetics and activation energy were evaluated from the growth of the deposited layer thickness. The apparent TEOS deposition activation energies were between −26.0 and 192 kJ mol^−1^ [35]. Simulation evaluation based on quantum chemistry was conducted for TEOS/O_3_ system [33,36]. The activated species of TEOS were proposed as Si(OH)(OC_2_H_5_)_3_ [37], O=Si(OC_2_H_5_)_2_ [38], or activated TEOS [34]. However, the counter diffusion CVD differs in diffusion and reaction of the nano-sized pore reactive species. Additionally, the organic substitutes are introduced into the chemical structure of the precursor, and the substituents are possibly decomposed depending on the deposition conditions. To optimize the performance of the silica membrane for gas separation, it is necessary to study the membrane formation conditions and optimize the deposition and decomposition reaction rates through several experimental investigations.

**Figure 2:**
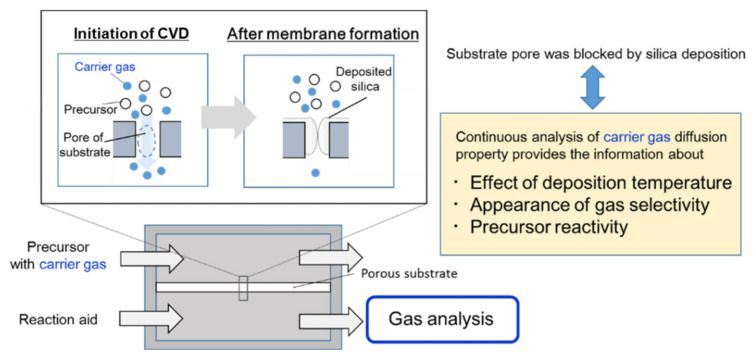
In situ analysis of counter diffusion CVD.

In this study, the counter diffusion CVD method was combined with gas analysis for the first time, and the diffusing properties of the carrier gas supplying the precursor were quantified continuously during CVD. This method is a novel evaluation method for membrane formation via counter diffusion CVD through the diffusion of the carrier gas. Figure 2 shows the schematic diagram of the CVD–gas analysis. The component of the outlet of the reaction aid side was introduced to mass spectroscopy with the analysis focused on the carrier gas used to supply precursors. At the beginning of deposition, carrier gas would diffuse to the opposite side through a porous substrate. As the deposition progressed, substrate pore closure by silica deposition reduced the diffusion amount of the carrier gas. This phenomenon was applied to investigate the effect of the experimental conditions, including the precursor structure and deposition temperature. The silica precursor’s effects were compared by using TMOS, TEOS, and HTMOS with mass spectroscopy imparting several mass-to-charge ratio (*m/z*) components. The decomposed component and the byproduct were investigated in terms of their compositions Moreover, the selectivity of the membrane prepared by multi-component bubbling was also monitored. 

## 2. Experimental

### 2.1. Preparation of γ-Alumina Substrate

The porous tuber α-alumina substrate (pore size: 150 nm; OD: 10 mm; ID: 7 mm; effective membrane length: 30 mm; effective membrane area: 9.42 cm^2^; NA−1, Noritake Co., Ltd., Aichi, Japan) was used as the substrate. The intermediate layer coated on α-alumina substrates was prepared using the same procedure as reported by Ishii et al. [18]. γ-Alumina sol was coated by using alumina sol (5S, Kawaken Fine Chemicals Co., Ltd., Tokyo, Japan) and pure water (1:1 [*vol/vol]*) solution. 30 mL of the mixture of alumina sol and pure water and 20 mL of a 3.5 wt% poly (vinyl alcohol) (*n* = 500, fully hydrolyzed; FUJIFILM Wako Pure Chemical Co., Osaka, Japan) aqueous solutions were mixed to obtain the coating sol. The porous tuber α-alumina substrate was immersed in the coating sol for 30 s. After drying at 60 °C for 3 h, the substrate was calcined at 600 °C for 3 h. The coating–calcination procedure was repeated twice.

### 2.2. Counter Diffusion CVD

**Figure 3:**
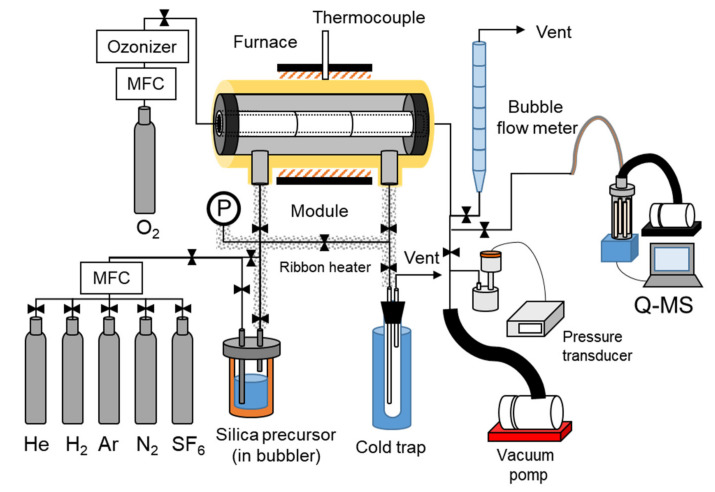
Counter diffusion CVD apparatus.

The silica membrane was prepared via the counter diffusion CVD method. Figure 3 shows the schematic diagram for the CVD apparatus, also used for measuring single gas permeation. The apparatus consists of the reactor module, gas feeding, trap, and permeation part. Both ends of the γ-alumina substrate were sealed by the graphite O-rings. The gas flow rates were controlled by using mass flow controllers (MFC, 1250RK, KOFLOC Corp., 3400, KOFLOC Corp., 8500, KOFLOC Corp., Kyoto, Japan). The silica precursor was kept in the bubbler bottle. First, the substrate was heated at 300–600 °C. The precursor vapor was supplied to the outer side of the tuber substrate by N_2_ or H_2_/N_2_ (50/50 [*v/v*%]) mixture as a carrier gas. The effects of the silica precursor were evaluated using TMOS, HTMOS, or TEOS. The partial vapor pressures of the precursor were controlled at 0.25–1.0 mol m^−3^ by controlling the bubbler temperature. Simultaneously, O_2_ was supplied to the inner side of the tuber substrate. The flow rates of the carrier gas and the oxidant were controlled at 200 mL min^−1^ and all the deposition was conducted under atmospheric pressure.

The outlet of the inner side of substrate was connected to the quadrupole mass spectroscopy (M201-QA-TDM, CANON ANELVA Corp.). Only O_2_ was introduced at the inlet of the inner side of the substrate. Thus, other molecules detected at the outlet of the inner substrate side diffused from the outer side of the substrate. 

### 2.3. Characterization

The gas passing through the inner side of the substrate was quantified using mass spectroscopy during CVD with a measurement conducted every 8 s. The analysis was performed in the range of *m*/*z* = 1 to *m*/*z* = 200, maintaining the ionization voltage at 30 eV. Here the mass detection intensity is proportional of the number of molecules. The intensity was corrected by the O_2_ intensity with *m*/*z* = 32, which is the carrier gas for the mass spectrometer via Equation (1) with the reference Hasegawa’s method [39]. The moving average deviation was kept *n* = 5 to decrease the noise effects.

(1) $$i-\mathrm{component}\;\mathrm{intensity}\;\mathrm{ratio}=\frac{m/z=n\;(=i-\mathrm{component}\;\mathrm{highest}\;\mathrm{peak})\;\mathrm{intensity}}{m/z=32\;\mathrm{intensity}}$$

The normalized detection amounts were calculated by dividing the maximum value during the CVD using Equation (2).

(2) $$\mathrm{Normalized}\;i-\mathrm{component}\;\mathrm{intensity}\;\mathrm{ratio}=\frac{m/z=n\;\mathrm{intensity}}{m/z=n\;\mathrm{maximum}\;\mathrm{intensity}}$$

In this paper, the intensity ratio for *m*/*z* = 28 was used to calculate the N_2_ concentration. The apparent kinetic constant was obtained by fitting with the time course change of the detection amount of the carrier gas of the initial deposition, and the apparent activation energy of precursor deposition reaction was estimated by using the apparent kinetic constant obtained for each condition.

The single gas permeation tests were conducted using H_2_ and N_2_ at the deposition temperature. The permeance was calculated Equation (3).

(3) $$P_{\;i}=\frac{F_{i}}{A\;\Delta p}\;$$

*P_i_* [mol m^−2^ s^−1^ Pa^−1^] is the permeance of component *i*, *F_i_* [mol s^−1^] is the flow rate of the permeated gas measured by bubble flow meter, *A* [m^−2^] is the membrane area, and the Δ*p* [Pa] is the pressure difference between the supply and the permeation side, measured by a pressure gauge. The ideal selectivity of the membrane was calculated by the ratio of gas permeance obtained by Equation (4).

(4) $$a_{i,\;j}=\frac{P_{i}}{P_{j}}\;$$

The cross-sectional view of the obtained membranes was observed using the scanning electron microscope (SEM), and the element distributions were analyzed by the energy dispersive X-ray spectrometer (EDS) (JSM-7610F, JEOL).

## 3. Results and Discussion

### 3.1. TMOS Deposition Analysis and Characterization

**Figure 4:**
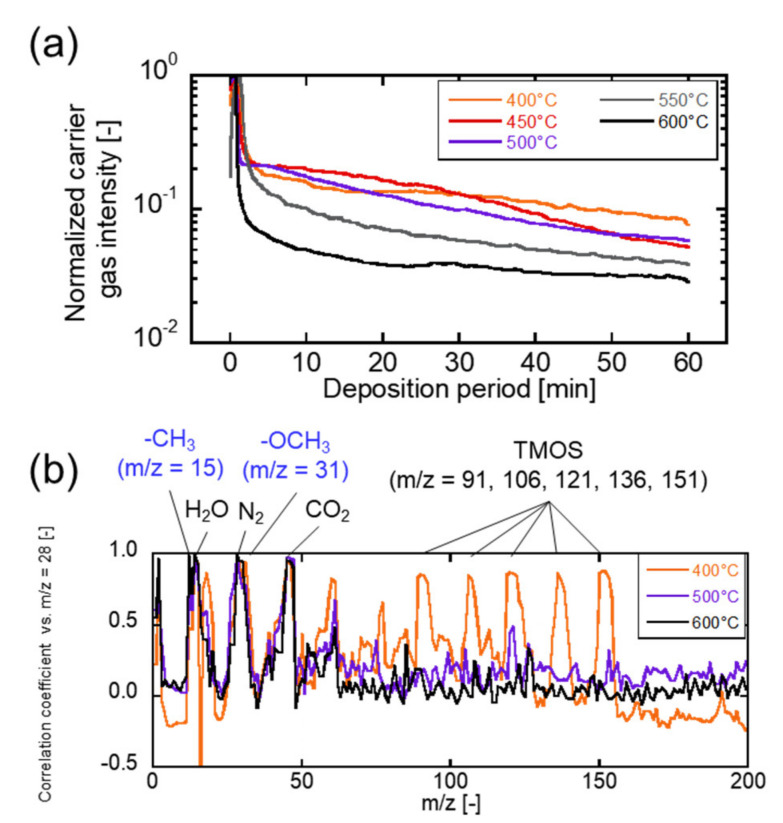
(**a**) The time course change of the detection of carrier gas (*m*/*z* = 28) during TMOS deposition. (**b**) The correlation coefficients of the time course changes of *m*/*z* = 1–200 and *m/z* = 28.

Figure 4a shows the time change of the detection of *m*/*z* = 28 during TMOS deposition. Depositions were conducted at 400–600 °C. N_2_ was used as the carrier gas. The detection of *m*/*z* = 28, which corresponds to the molecular weight of N_2_, showed the highest change among the detected *m*/*z*. The maximum value was detected at 50 s after starting the deposition, the intensity decreased exponentially, with the value decreased less than 1/10 within 5 min in all deposition conditions. The time change was gradually decreased, and later stabilized. Deposition temperature affected the first *m*/*z* = 28 detection decreasing rate. The deposition reaction rate affected the diffusion amount of the carrier gas. The decreasing rate represented the deposition reaction rate indirectly. Figure 4b shows the correlation coefficient of the time course change of *m*/*z* = 1–200 and that of *m*/*z* = 28 detected during the TMOS deposition. The higher values indicated the decomposed precursor components or byproduct-derived deposition reaction, diffusing with the carrier gas simultaneously. The *m*/*z* = 1, 15, 17, 18, 44, 45 47, 59, 61, 91, 106, 121, 136, and 151 were strongly correlated during the TMOS deposition. Especially the combination of *m*/*z* = 91, 106, 121, 136, and 151 were the mass spectrum-derived TMOS. The unreacted TMOS diffused to the reaction aid side and was detected at 400 °C CVD. Those mass spectrum correlations were not observed during the 500 and 600 °C CVD. The *m*/*z* = 1, 15, 17, 18, 44, 45 47, 59, and 61 were detected for all depositions. The fragments suggested methoxy (-OCH_3_) from *m*/*z* = 15 and 31, water (H_2_O) from *m*/*z* = 1, 17, and 18, carbon dioxide (CO_2_) from *m*/*z* = 44, and decompositions were derived from the precursor. The *m*/*z* = 45, 47, 59, and 61 were measured at all deposition temperatures. At 500 °C and 600 °C, there was no correlation with the TMOS-derived peak at *m*/*z* = 121, suggesting that those detections are not a fragment of the precursor due to its decomposition in the mass spectrometer, but possibly a decomposition product or fragment of a product resulting from the reaction. The chemical formulas guessed from the reaction molecular weights (MWs) were OSiH (MW: 45), OSiH_3_(MW: 47), SiOCH_3_ (MW: 59), and H_2_SiOCH_3_ (MW: 61) including radical species, and the structural features were the decomposed alkoxy groups in the precursor. Therefore, the decomposition of alkoxy groups could be involved in the deposition reaction.

**Figure 5:**
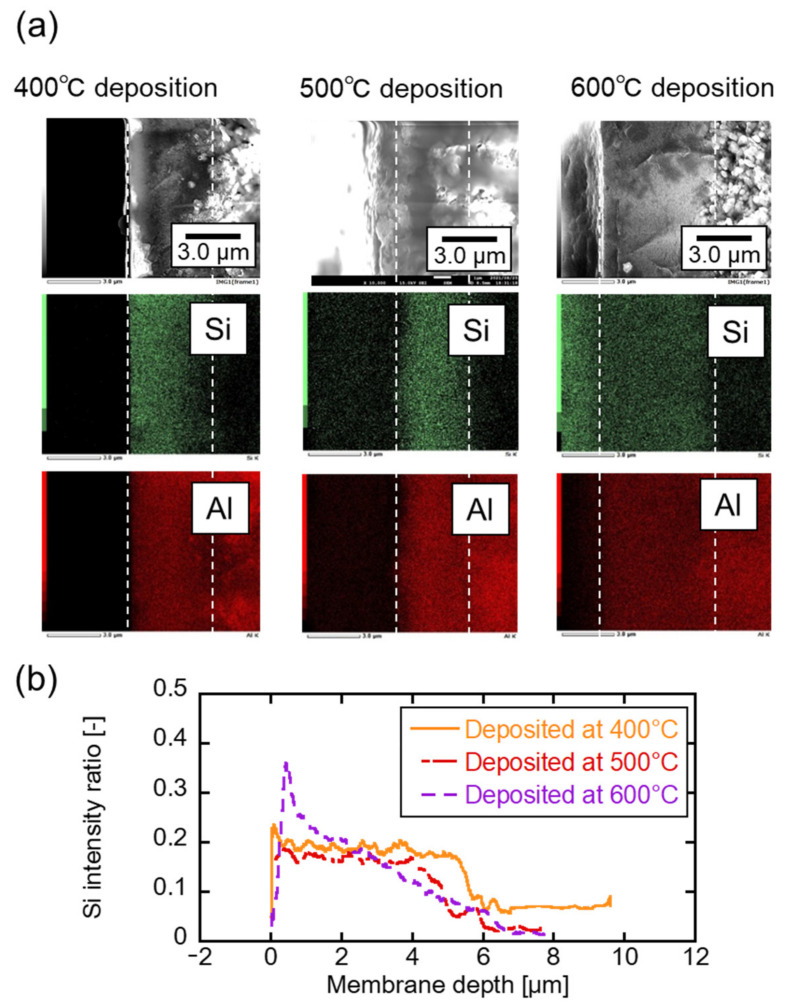
(**a**) Cross-sectional views and elemental mapping of the TMOS-derived membrane, (**b**) Si distribution of the direction of the membrane depth.

Figure 5a shows the SEM and EDS images of the cross-sectional view of the membrane prepared at 400, 500, and 600 °C and the elemental mapping results. The left and right sides are the outer and inner parts of the membrane. A smooth layer is observed in the center in the SEM image, which is the γ-alumina layer coated on the substrate. The particle layer was at the inner side of the membrane, the α-alumina porous substrate. The thickness of the γ-alumina interlayer was observed as 5–6 μm, although it appeared differently because of the high measurement magnification. The intermediate layer thickness does not reportedly affect the reaction since the reactive species diffuse in a fraction of a second in CVD. The existence of aluminum (Al) was observed in the support and the intermediate layer by elemental mapping. Silicone (Si) derived from CVD silica was observed mainly in the γ-alumina layer at all deposition temperatures. The Si detection inside the γ-alumina layer was confirmed in previous deposition studies as well [26,40,41]. Line analysis was conducted to evaluate the silica layer distribution. Figure 5b shows the ratio of the Si detected in the depth direction as 0.2. The membrane prepared at 400 °C deposited the Si homogenously. As the deposition temperature increased, Si was detected near the membrane surface. In counter diffusion CVD, the reaction species diffused to the porous substrate. The deviation implies the precursor reaction rate faster than the diffusion rate at a high-temperature deposition. 

**Figure 6:**
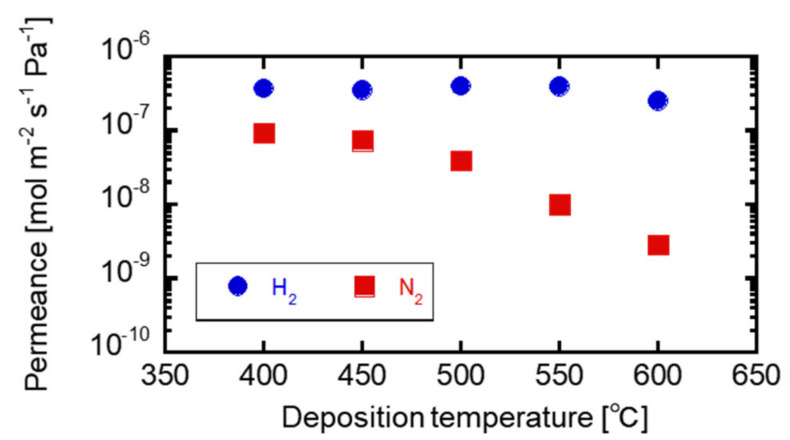
The TMOS-derived membrane gas performances at each deposition temperature.

Figure 6 shows the gas permeation performance of the TMOS-derived membrane. At a higher deposition temperature, the N_2_ gas permeance decreased, and the H_2_/N_2_ gas permeance ratio increased. At 400 and 450 °C depositions, the H_2_/N_2_ gas permeance ratios were 4.0 and 4.8, respectively, and close to the Knudsen ratio of 3.7. At 500, 550, and 600 °C depositions, the H_2_/N_2_ gas permeance ratios obtained were 10, 40, and 88. The H_2_ permeance almost remained unchanged with the change in the deposition temperature. At 600 °C deposition, the membrane showed the H_2_ gas permeance of 2.5–4.0 × 10^−7^ mol m^−2^ s^−1^ Pa^−1^. The membrane formation easily occurred due to high deposition rate and pore closure. 

### 3.2. Correlation between the Membrane Perfomance and Carrier Gas Detection, and Applying Multicomponent Carrier to Evaluate Membrane Formation

**Figure 7:**
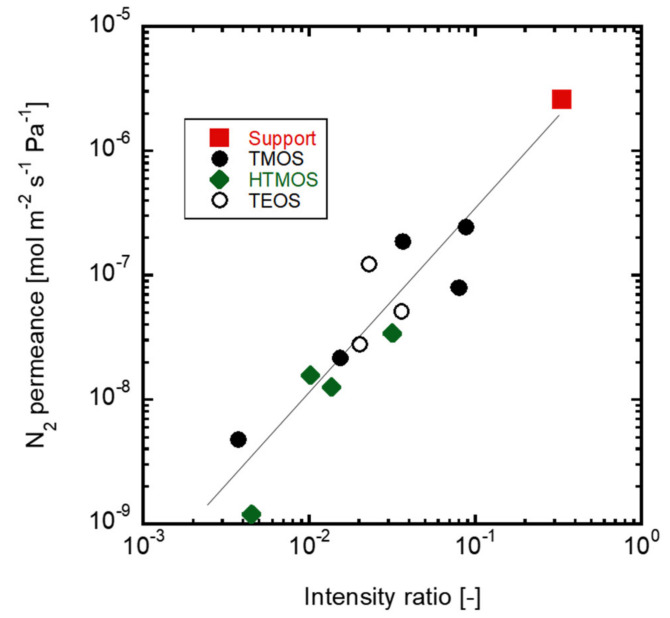
The relationships between the detections of *m/z* = 28 at the end of CVD and N_2_ gas permeance of obtained membranes.

Figure 7 shows the relationship between the *m/z* = 28 intensities at the end of CVD and the N_2_ permeances of the obtained membrane. The intensities obtained by the mass spectrometer and the permeance obtained by the single gas permeation tests agreed with each other in a wide range of 10^−6^ to 10^−9^ mol m^−2^ s^−1^ Pa^−1^. The difference in the silica precursors was negligible showing the effects of the deposited silica on N_2_ diffusion. The permeation performance of the CVD silica membranes during deposition could be evaluated by sampling the outlet gas with mass spectroscopy. The diffusion amounts of multiple components were further evaluated, and the separation performance was observed. Using alkoxysilane deposition, the reaction automatically stops due to the suppression of the diffusion of the silica precursor caused by the deposited silica on the substrate during the counter diffusion CVD method [14]. However, the reaction should be stopped before the pores of the substrates are filled with the silica in the case of alkylalkoxysilane to obtain larger pores. If the target molecules, such as H_2_ or hydrocarbons are introduced in the carrier gases, the deposition period would be decided by monitoring the diffusion of the target molecule during CVD using any precursor in any situation.

**Figure 8:**
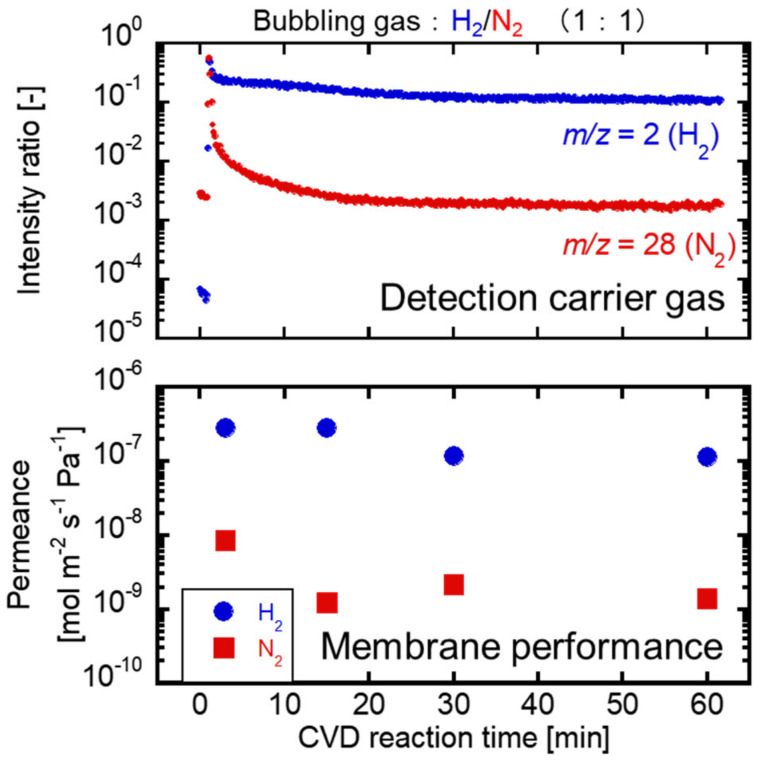
The diffusion properties of binary carrier gases and membrane performance in TMOS deposition at 550 °C.

Figure 8 shows the time course of the intensities of H_2_ (*m/z* = 2) and N_2_ (*m/z* = 28) during TMOS deposition at 550 °C and the H_2_ and N_2_ permeances measured by the single gas permeation tests. The TMOS vapor feeding was conducted by H_2_/N_2_ bubbling. The maximum *m/z* intensities of H_2_ and N_2_ were detected immediately after introducing the carrier gases into the membrane module. N_2_ intensities decreased sharply to less than 1% of the initial intensity within 6 min, while those of H_2_ decreased to only 40%. Even when compared with the actual permeation test results, *m/z* and gas permeance corresponded to the results shown in Figure 7. The intensity of N_2_ showed the real time deposition progress. The effects of H_2_ in the carrier gases on the deposition of TMOS/O_2_ were unclear; however, the H_2_ permselective membranes were obtained. The H_2_/N_2_ permeance ratios were 33, 220, and 107 through the membrane deposited at 3 min, 15 min, and 30 min, respectively. The membrane deposited at 60 min showed an H_2_ permeance of 1.2 × 10^−7^ mol m^−2^ s^−1^ Pa^−1^ with the H_2_/N_2_ permeance ratio of 86. 15 min deposition was enough to obtain the H_2_ permselective membrane at 550 °C. The separation performance was slightly reduced after 15 min deposition. Regarding the subsequent deterioration of performance, the silica precursor was deposited on the surface by autolysis in the high-temperature deposition condition. O_2_ diffusion should rarely occur due to the reduction of N_2_ permeance through the membrane. 

Generally, the membrane gas permeation performance evaluation is conducted after membrane preparation [23]. In this novel method, the amount of carrier gas diffusion could be monitored using mass spectrometry, therefore decreasing gas permeance through the diffusion of carrier gas during CVD. If the carrier gas was mixed with the molecules to be separated analyzing the diffusion properties, it would be easy to optimize the deposition period to determine whether the separation performance of the molecules to be separated was based on the change in the diffusion amount. In addition, by using gases with various molecular sizes as the carrier gases, it is possible to evaluate the pore formation process of CVD silica membranes.

### 3.3. The Evaluation of the Kinetics of Precursor by Using Carrier Gas Diffusion

**Figure 9:**
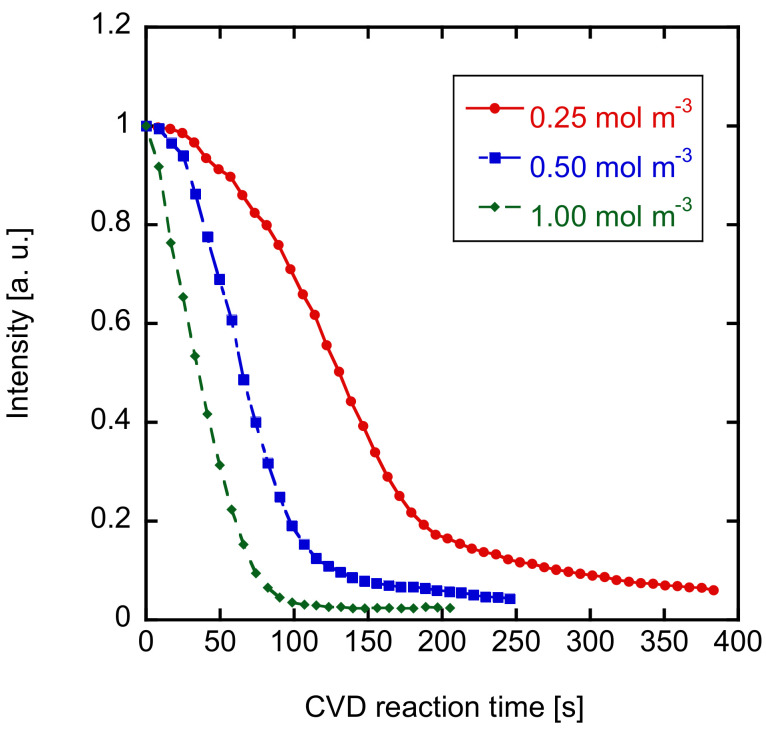
Effects of the precursor concentration of the decreasing trend of the detection of carrier gas at 450 °C HTMOS deposition.

The time courses of the initial intensities of N_2_ should be related to the deposition rate in the substrate pores. The deposition rates were discussed by changing the silica precursors. First, the effects of the vapor pressure of HTMOS were investigated. Figure 9 shows the time course of the intensities for the HTMOS deposition at 450 °C. The maximum intensities of N_2_ were defined as t = 0 min. The vapor pressures of HTMOS were kept at 0.25, 0.50, or 1.0 mmol L^−1^. The intensity decreased sharply at higher HTMOS vapor pressures. The half-life periods of the intensities were 130, 63, and 33 s for the HTMOS vapor pressures of 0.25, 0.50, and 1.0 mmol L^−1^, respectively. The half-life periods of the intensities and the HTMOS vapor pressures were inversely proportional showing that the apparent deposition reaction is a first-order reaction. 

**Figure 10:**
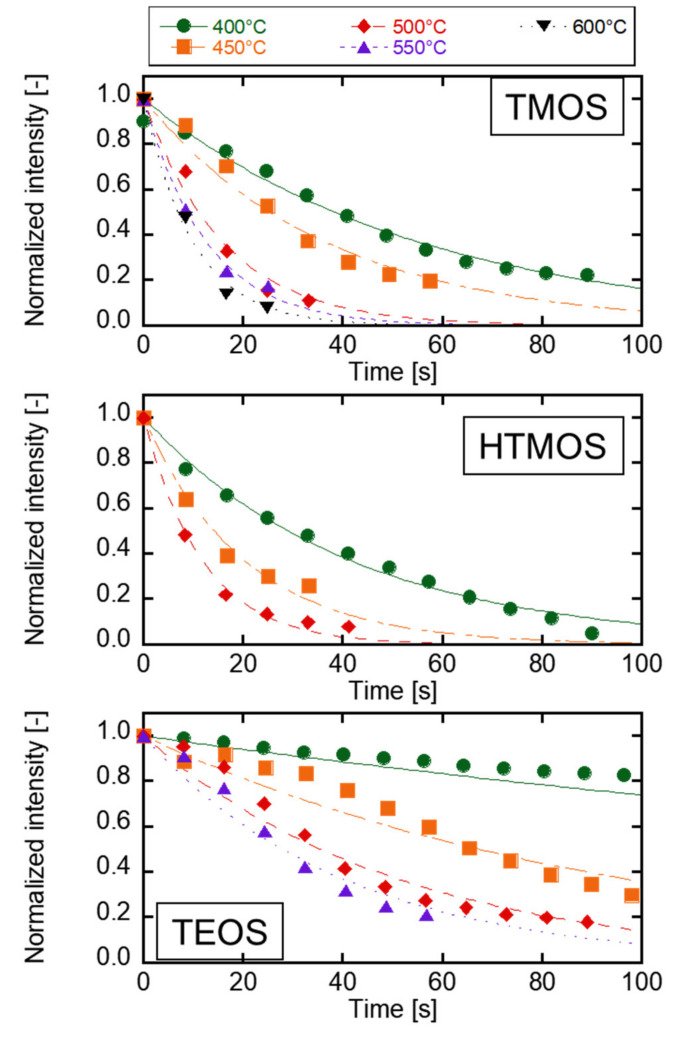
Time course change in the detection of carrier gas components in initial CVD (**Upper**) TMOS deposition, (**Middle**) HTMOS deposition, and (**Lower**) TEOS deposition.

Figure 10 shows the time course of the normalized intensities of N_2_ by changing the precursors. All the normalized intensities decreased exponentially, and the intensities were less than 10% of the initial values within 100 s. The half-life periods of the intensities increased with the increasing deposition temperature. The HTMOS deposition rate was faster than that of the TMOS. The probability of adhesion to the substrate may be different for TMOS and HTMOS. The TEOS deposition rates were obviously slower than those of the TMOS and HTMOS. The main structural difference between TEOS and TMOS was the alkoxy group. The decomposition reaction was explained via Si-OH or Si=O [37,38] from the detection of the fragments *m/z* and the difference in the precursor structures. The decomposition of alkoxy groups, such as methoxy or ethoxy groups in the structure should be affected in CVD reaction.

**Figure 11:**
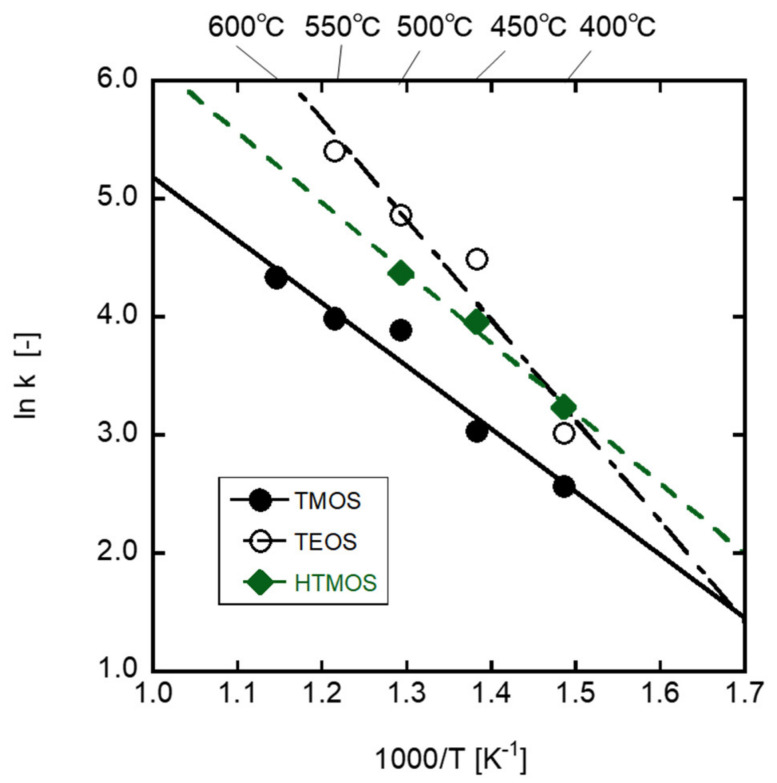
Arrhenius plot of the apparent deposition rate using each precursor.

As discussed in the previous section, the apparent deposition reaction of the silica precursor seemed a first-order reaction. Here, the apparent rate constants were calculated from the intensities of N_2_ within the first 2 min. The deposition kinetics controlled the membrane performances for various separation systems. Figure 11 shows the Arrhenius plots of the apparent rate constants. The activation energies of the deposition reaction can be calculated by the slopes of the plots. The activation energies of TMOS, HTMOS, and TEOS decompositions were 44.3 kJ mol^−1^, 49.4 kJ mol^−1^, and 71.0 kJ mol^−1^, respectively. The activation energy of TEOS decomposition was much larger than those of TMOS and HTMOS. As discussed in the former section, the difference should be attributed to the alkoxy groups of the precursors. Thus, the activation energies calculated from Figure 11 must be the decomposition reaction of alkoxy groups of the precursors. The deposition reaction of the CVD silica membrane involved the reaction derived from the alkoxy group and affected the deposition rates. Alkoxy groups for the precursors, vapor pressure, and deposition temperature are important parameters for vapor deposition control in silica membrane development.

## 4. Conclusions

The membrane formation behavior of the counter diffusion CVD method was analyzed by measuring the diffusion properties of carrier gas through the porous substrates during the CVD. The detected amount of carrier gas was attributed to the membrane permeance. The analysis provided new insights, such as decomposing products, membrane selectivity, and precursor reactivity to develop gas separation membranes using the CVD method. The separation behavior was successfully evaluated by using multi-component carrier gases. The amount of N_2_ diffusion decreased immediately after the start of the deposition, and a membrane with an H_2_/N_2_ gas permeance ratio of more than 100 was obtained with TMOS deposition. The deposition properties were compared by changing the silica precursors among TMOS, TEOS, and HTMOS. The deposition rates increased by changing the deposition temperature. The apparent activation energies of the deposition reaction were 44.3, 49.4, and 71.0 kJ mol^−1^ for TMOS, TEOS, and HTMOS deposition, respectively. Alkoxy groups for the precursors, vapor pressure, and deposition temperature are important parameters for vapor deposition control in silica membrane development.

## References

[1] Myagmarjav O., Tanaka N., Nomura M., Kubo S. (2017). Hydrogen production tests by hydrogen iodide decomposition membrane reactor equipped with silica-based ceramics membrane. Int. J. Hydrogen Energy.

[2] Myagmarjav O., Iwatsuki J., Tanaka N., Noguchi H., Kamiji Y., Ioka I., Kubo S., Nomura M., Yamaki T., Sawada S. (2019). Research and development on membrane IS process for hydrogen production using solar heat. Int. J. Hydrogen Energy.

[3] Kusakabe K., Kuroda T., Morooka S. (1998). Separation of carbon dioxide from nitrogen using ion-exchanged faujasite-type zeolite membranes formed on porous support tubes. J. Memb. Sci..

[4] Sakai M., Sasaki Y., Tomono T., Seshimo M., Matsukata M. (2019). Olefin Selective ag-exchanged X-type zeolite membrane for propylene/propane and ethylene/ethane separation. ACS Appl. Mater. Interfaces.

[5] Kusakabe K., Sakamoto S., Saie T., Morooka S. (1999). Pore structure of silica membranes formed by a sol-gel technique using tetraethoxysilane and alkyltriethoxysilanes. Sep. Purif. Technol..

[6] Kanezashi M., Yada K., Yoshioka T., Tsuru T. (2010). Organic-inorganic hybrid silica membranes with controlled silica network size: Preparation and gas permeation characteristics. J. Memb. Sci..

[7] Hwang G. (1999). Hydrogen separation in H_2_–H_2_O–HI gaseous mixture using the silica membrane prepared by chemical vapor deposition. J. Memb. Sci..

[8] Gavalas G.R., Megiris C.E., Nam S.W. (1989). Deposition of H_2_-permselective SiO_2_ films. Chem. Eng. Sci..

[9] Okubo T., Inoue H. (1989). Introduction of specific gas selectivity to porous glass membranes by treatment with tetraethoxysilane. J. Memb. Sci..

[10] Yamaguchi T., Ying X., Tokimasa Y., Nair B.N., Sugawara T., Nakao Q. (2000). Reaction control of tetraethyl orthosilicate (TEOS)/O_3_ and tetramethyl orthosilicate (TMOS)/O_3_ counterdiffusion chemical vapour deposition for preparation of molecular-sieve membranes. Phys. Chem. Chem. Phys..

[11] Nagasawa H., Shigemoto H., Kanezashi M., Yoshioka T., Tsuru T. (2013). Characterization and gas permeation properties of amorphous silica membranes prepared via plasma enhanced chemical vapor deposition. J. Memb. Sci..

[12] Nagasawa H., Yamamoto Y., Tsuda N., Kanezashi M., Yoshioka T., Tsuru T. (2017). Atmospheric-pressure plasma-enhanced chemical vapor deposition of microporous silica membranes for gas separation. J. Memb. Sci..

[13] Lee D., Oyama S.T. (2002). Gas permeation characteristics of a hydrogen selective supported silica membrane. J. Memb. Sci..

[14] Nomura M., Ono K., Gopalakrishnan S., Sugawara T., Nakao S.I. (2005). Preparation of a stable silica membrane by a counterdiffusion chemical vapor deposition method. J. Memb. Sci..

[15] Nakao S.I., Suzuki T., Sugawara T., Tsuru T., Kimura S. (2000). Preparation of microporous membranes by TEOS/O_3_ CVD in the opposing reactants geometry. Microporous Mesoporous Mater..

[16] Nomura M., Seshimo M., Aida H., Nakatani K., Gopalakrishnan S., Sugawara T., Ishikawa T., Kawamura M., Nakao S.I. (2006). Preparation of a catalyst composite silica membrane reactor for steam reforming reaction by using a counterdiffusion CVD method. Ind. Eng. Chem. Res..

[17] Ahn S.J., Yun G.N., Takagaki A., Kikuchi R., Oyama S.T. (2018). Effects of pressure, contact time, permeance, and selectivity in membrane reactors: The case of the dehydrogenation of ethane. Sep. Purif. Technol..

[18] Ishii K., Nagataki Y., Yoshiura J., Saito Y., Nagataki T., Nomura M. (2021). Development of hydrogen permselective membranes for propylene production. J. Chem. Eng. Jpn..

[19] Oda K., Akamatsu K., Sugawara T., Kikuchi R., Segawa A., Nakao S.I. (2010). Dehydrogenation of methylcyclohexane to produce high-purity hydrogen using membrane reactors with amorphous silica membranes. Ind. Eng. Chem. Res..

[20] Sea B.K., Kusakabe K., Morooka S. (1997). Pore size control and gas permeation kinetics of silica membranes by pyrolysis of phenyl-substituted ethoxysilanes with cross-flow through a porous support wall. J. Memb. Sci..

[21] Ohta Y., Akamatsu K., Sugawara T., Nakao A., Miyoshi A., Nakao S.I. (2008). Development of pore size-controlled silica membranes for gas separation by chemical vapor deposition. J. Memb. Sci..

[22] Nomura M., Nagayo T., Monma K. (2007). Pore size control of a molecular sieve silica membrane prepared by a counter diffusion CVD method. J. Chem. Eng. Jpn..

[23] Ishii K., Shibata A., Takeuchi T., Yoshiura J., Urabe T., Kameda Y., Nomura M. (2019). Development of silica membranes to improve dehydration reactions. J. Jpn. Pet. Inst..

[24] Ikeda A., Nomura M. (2016). Preparation of amorphous silica based membranes for separation of hydrocarbons. J. Jpn. Pet. Inst..

[25] Matsuyama E., Ikeda A., Komatsuzaki M., Sasaki M., Nomura M. (2014). High-temperature propylene/propane separation through silica hybrid membranes. Sep. Purif. Technol..

[26] Myagmarjav O., Ikeda A., Tanaka N., Kubo S., Nomura M. (2017). Preparation of an H_2_-permselective silica membrane for the separation of H_2_ from the hydrogen iodide decomposition reaction in the iodine–sulfur process. Int. J. Hydrogen Energy.

[27] Myagmarjav O., Tanaka N., Nomura M., Kubo S. (2019). Module design of silica membrane reactor for hydrogen production via thermochemical IS process. Int. J. Hydrogen Energy.

[28] Ikeda A., Matsuyama E., Komatsuzaki M., Sasaki M., Nomura M. (2014). Development of inorganic silica reverse osmosis membranes by using a counter diffusion chemical vapor deposition method. J. Chem. Eng. Jpn..

[29] Ishii K., Ikeda A., Takeuchi T., Yoshiura J., Nomura M. (2019). Silica-based ro membranes for separation of acidic solution. Membranes.

[30] Yoshiura J., Ishii K., Saito Y., Nagataki T., Nagataki Y., Ikeda A., Nomura M. (2020). Permeation properties of ions through inorganic silica-based membranes. Membranes.

[31] Hong L.S., Shimogaki Y., Komjyama H. (2000). Macro/microcavity method and its application in modeling chemical vapor deposition reaction systems. Thin Solid Films.

[32] Ponton S., Vergnes H., Samelor D., Sadowski D., Vahlas C., Caussat B. (2018). Development of a kinetic model for the moderate temperature chemical vapor deposition of SiO_2_ films from tetraethyl orthosilicate and oxygen. AIChE J..

[33] Ponton S., Dhainaut F., Vergnes H., Samelor D., Sadowski D., Rouessac V., Lecoq H., Sauvage T., Caussat B., Vahlas C. (2019). Investigation of the densification mechanisms and corrosion resistance of amorphous silica films. J. Non-Cryst. Solids.

[34] Kim E.J., Gill W.N. (1994). Modeling of CVD of Silicon Dioxide Using TEOS and Ozone in a Single-Wafer Reactor. J. Electrochem. Soc..

[35] Pavelescu C., Kleps I. (1990). Activation energies in chemical vapour deposition kinetics of SiO_2_ films using TEOS chemistry. Thin Solid Films.

[36] Kim E.J., Gill W.N. (1994). Analytical model for chemical vapor deposition of SiO_2_ films using tetraethoxysilane and ozone. J. Cryst. Growth.

[37] Kawahara T., Yuuki A., Matsui Y. (1992). Reaction mechanism of chemical vapor deposition using tetraethylorthosilicate and ozone at atmospheric pressure. Jpn. J. Appl. Phys..

[38] Sirakami K., Kobayashi K., Kikuchi H., Fuwa A. (1996). Thermal decomposition of tetraethoxysilane studied by atmospheric mass-spectrometer. Nippon Kinzoku Gakkaishi.

[39] Hasegawa Y., Kimura K., Nemoto Y., Nagase T., Kiyozumi Y., Nishide T., Mizukami F. (2008). Real-time monitoring of permeation properties through polycrystalline MFI-type zeolite membranes during pervaporation using mass-spectrometry. Sep. Purif. Technol..

[40] Araki S., Mohri N., Yoshimitsu Y., Miyake Y. (2007). Synthesis, characterization and gas permeation properties of a silica membrane prepared by high-pressure chemical vapor deposition. J. Memb. Sci..

[41] Park H.M., Lee J.Y., Jee K.Y., Nakao S.i., Lee Y.T. (2021). Hydrocarbon separation properties of a CVD-deposited ceramic membrane under single gases and binary mixed gas. Sep. Purif. Technol..

